# Modified Nanoemulsions with Iron Oxide for Magnetic Resonance Imaging

**DOI:** 10.3390/nano6120223

**Published:** 2016-11-25

**Authors:** Yongyi Fan, Rui Guo, Xiangyang Shi, Steven Allen, Zhengyi Cao, James R. Baker, Su He Wang

**Affiliations:** 1 Michigan Nanotechnology Institute for Medicine and Biological Sciences and Department of Internal Medicine, University of Michigan, Ann Arbor, MI 48109, USA; yyfan@umich.edu (Y.F.); zhengyi@umich.edu (Z.C.); jbakerjr@umich.edu (J.R.B.); 2 College of Chemistry, Chemical Engineering and Biotechnology, Donghua University, Shanghai 201620, China; ruiguo@dhu.edu.cn (R.G.); xshi@dhu.edu.cn (X.S.); 3 Biomedical Engineering, University of Michigan, 2131 Gerstacker, 2200 Bonisteel Blvd., Ann Arbor, MI 48105, USA; stevepa@umich.edu

**Keywords:** nanoemulsion, imaging, iron oxide, tracking

## Abstract

A nanoemulsion (NE) is a surfactant-based, oil-in-water, nanoscale, high-energy emulsion with a mean droplet diameter of 400–600 nm. When mixed with antigen and applied nasally, a NE acts as a mucosal adjuvant and induces mucosal immune responses. One possible mechanism for the adjuvant effect of this material is that it augments antigen uptake and distribution to lymphoid tissues, where the immune response is generated. Biocompatible iron oxide nanoparticles have been used as a unique imaging approach to study the dynamics of cells or molecular migration. To study the uptake of NEs and track them in vivo, iron oxide nanoparticles were synthesized and dispersed in soybean oil to make iron oxide-modified NEs. Our results show that iron oxide nanoparticles can be stabilized in the oil phase of the nanoemulsion at a concentration of 30 µg/μL and the iron oxide-modified NEs have a mean diameter of 521 nm. In vitro experiments demonstrated that iron oxide-modified NEs can affect uptake by TC-1 cells (a murine epithelial cell line) and reduce the intensity of magnetic resonance (MR) images by shortening the T2 time. Most importantly, in vivo studies demonstrated that iron oxide-modified NE could be detected in mouse nasal septum by both transmission electron microscopy and MR imaging. Altogether these experiments demonstrate that iron oxide-modified NE is a unique tool that can be used to study uptake and distribution of NEs after nasal application.

## 1. Introduction

Nasal vaccine administration has been used as an alternative route for vaccination. Mucosal adjuvants are substances that are combined with antigens in vaccines to enhance the humoral and/or cell-mediated immune responses. Oil-in-water nanoemulsion (NE) is a nanoscale (mean droplet size of less 400 nm in diameter) emulsion formulated with ionic and nonionic surfactants, soybean oil, ethanol and water using a high speed homogenizer [1,2]. While NE was originally developed as a microbicidal agent, subsequent studies fortuitously demonstrated that NE formulations are also highly effective as a mucosal adjuvant in nasopharyngeal vaccines [3,4,5,6,7,8,9,10,11,12,13,14].

A number of publications have demonstrated that nasal immunization with NE-coated recombinant antigens or inactive viruses are able to elicit not only a local mucosal immune response but also a systemic immune response, producing high levels of protective immunity against infections with influenza, anthrax or viruses [3,4,5,6,7,8,9,10,11,13]. Although it is known that NE as a mucosal adjuvant can facilitate antibody production, Th1/Th2 and Th17 cell responses, regulatory T cell induction, cytokine secretion, dendritic cell engulfment and trafficking, the mechanisms responsible for this robust response are far from clear [3,8]. Recent experiments in mice have suggested that antigens coated by NE and administered nasally may travel to the thymus [15], a critical immune organ where T-lymphocytes develop. However, a reliable and stable tracking/labelling tool is not yet available for NE, which hampers mechanistic studies and the further development of NE-related vaccines.

Biocompatible iron oxide nanoparticles have been used for various biomedical applications where they facilitate laboratory diagnostics and therapeutics. Recent studies have demonstrated that biocompatible iron oxide-based tracking systems can be used as diagnostic agents in magnetic resonance (MR) imaging as well as for drug delivery vehicles in vivo [16,17,18,19]. Based on these studies, we hypothesized that combining NE with iron oxide nanoparticles may make it possible to track the uptake and migration of NE in vivo. To test this we modified iron oxide nanoparticles (Fe_3_O_4_) using surface coating in order to disperse them into soybean oil, and then used this mixture to produce and characterize iron oxide-modified NE (Fe_3_O_4_ NE). Finally, experiments were conducted to determine whether this oxide-modified NE can readily be located and stably tracked in vivo.

## 2. Results and Discussion

**Figure 1:**
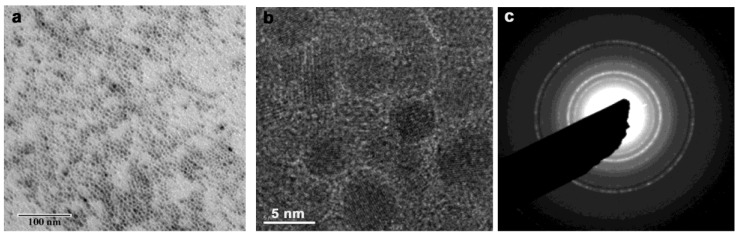
(**a**) Transmission electron micrograph; (**b**) High-resolution transmission electron micrograph; (**c**) Selected area electron diffraction pattern of Fe_3_O_4_ nanoparticles.

In the present study we first prepared iron oxide nanoparticles through thermo-decomposition of Fe(acac)_3_ in phenyl ether with oleic acid and oleylamine, according to a well-established organic phase synthesis procedure [20]. The resulting Fe_3_O_4_ nanoparticles were subsequently purified by precipitation and redispersion of the nanoparticles using ethanol and hexane respectively. According to transmission electron microscopy (TEM) result (Figure 1a), the resultant iron oxide-nanoparticles appeared as very uniform, short-range hexagonal arrays, and the size was around 4.2 nm, which is consistent with a previous report [20]. The high-resolution TEM image in Figure 1b clearly shows the crystallinity of Fe_3_O_4_ NPs. The interplanar distance measured from the adjacent lattice fringes is about 0.26 nm, corresponding to the (311) planes of Fe_3_O_4_ NPs [21]. The selected area electron diffraction (SAED) pattern of Fe_3_O_4_ (Figure 1c) exhibits bright and distinguishable diffraction rings, suggesting the high crystallinity of Fe_3_O_4_ NPs. The diffraction rings can be indexed to the (311), (400), (422), (511) and (440) lattice planes, demonstrating the formation of the face-centered cubic (fcc) structure of Fe_3_O_4_ NPs. The lattice spacing, measured based on the diffraction rings, is in accordance with the standard lattice spacing of Fe_3_O_4_ from the PDF database [20,22]. Therefore, oil-stabilized iron oxide nanoparticles have been synthesized successfully.

**Scheme 1:**
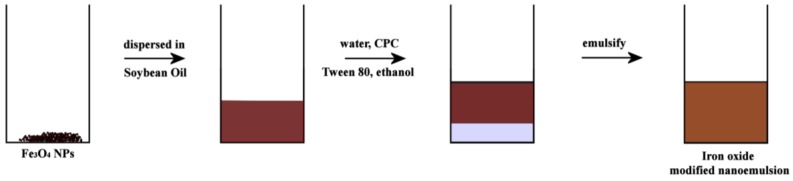
Schematic illustration of the synthesis of iron oxide-modified nanoemulsions (NEs).

To make an MRI-trackable nanoemulsion, iron oxide nanoparticles were dispersed in soybean oil as a homogenous solution. Then nanoemulsion was prepared by a previously described method with the components of Fe_3_O_4_-soybean oil, water, cetylpyridinium chloride (CPC), Tween 80 and ethanol, as shown in Scheme 1 [14,15]. As shown in Figure S1, when the concentration of iron oxide in soybean oil increased to 40 µg/µL, the iron oxide-modified NE was unstable and phase separation appeared after storage. Hence, in this study, the concentration of iron oxide is set at 30 µg/µL to get a balance between the stability and the MRI signal efficiency of iron oxide-modified NEs. The final concentration of Fe in nanoemulsion was measured by inductively-coupled plasma-high resolution mass spectrometry (ICP-MS) as 1.175 ± 0.06 µg/µL. The average hydrodynamic diameter of iron oxide-modified NE is 521.1nm, while the poly-dispersity index is 0.305, and the zeta potential is 9.64 ± 0.755 mV as characterized by a zetasizer (Malvern Instruments Ltd. Malvern, UK). Moreover, similar to the pure NE, the formed iron oxide-modified NE remains as an oil-in-water nanoscale emulsion with good stability by storing at 4 °C.

With around 520 nm in size, the iron oxide modified NEs are likely to enter the cells mainly by phagocytosis and less frequently by permeability. There are reports that the cells can uptake 0.5–8 µm particles by phagocytosis [23]. Particles around 500 nm can also enter the cytosol via cellular permeability [24]. It is known that particles bigger than 200 nm are usually filtered by the liver, while smaller particles can be quickly cleared by the kidney or through extravasation [25]. Therefore, we believe the liver may be the main organ that degrades the iron oxide-modified NEs tested.

**Figure 2:**
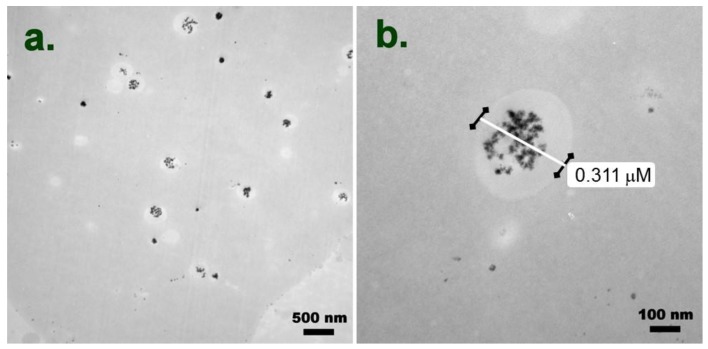
TEM images of iron oxide-modified NE in histo-gel at (**a**) 19,000×; and (**b**) 130,000×.

TEM imaging of histogel that encapsulated iron oxide-modified NE clearly demonstrates that iron oxide was located inside NE droplets (Figure 2). The majority of iron oxide-modified NE was found to contain multiple iron oxide cores, which were frequently aggregated into larger clusters (Figure 2b). The distribution of Fe_3_O_4_ NPs in nanoemulsion is further demonstrated by analysing the Fe concentration in different phases of iron oxide modified nanoemulsion by ICP-MS (Figure S2). It is demonstrated that only about 1% of iron oxide nanoparticles were found in the aqueous phase, whereas about 64% were located in the oil phase and 35% in interphase. Therefore, the iron oxide stabilized in nanoemulsion could effectively illustrate the movement of nanoemulsion in the body as MRI agents.

### 2.1. Cytotoxicity Assay and Cellular Uptake Assay

**Figure 3:**
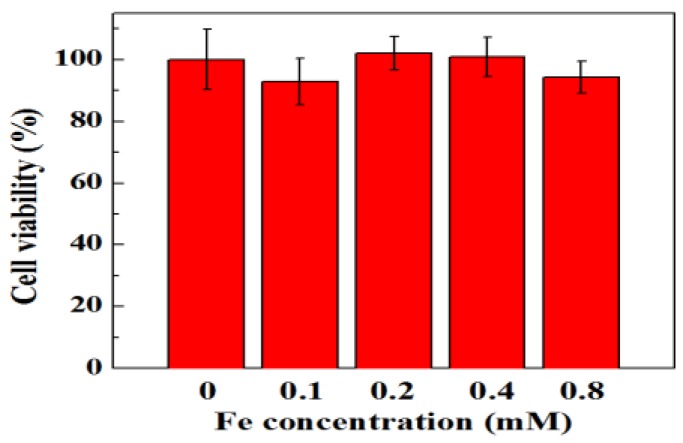
2,3-Bis-(2-methoxy-4-nitro-5-sulfophenyl)-2*H*-tetrazolium-5-carboxanilide (XTT) viability assay of TC-1 cells treated with Fe_3_O_4_ modified NEs at Fe concentration of 0*–*0.8 mM for 15 min. TC-1 cells treated with phosphate-buffered saline (PBS) were used as control.

In this study, TC-1 cells were used as a model cell line. 2,3-bis-(2-methoxy-4-nitro-5-sulfophenyl)-2*H*-tetrazolium-5-carboxanilide (XTT) assay is used to evaluate the viability of TC-1 cells incubated with NEs at Fe concentrations up to 0.8 mM for 15 min (Figure 3). It can be clearly seen that the viability of TC-1 cells remains more than 90%, indicating that NEs show no appreciable cytotoxicity. The non-cytotoxic nature of NE coupled with iron oxide is in line with previous data showing highly biocompatibility of NE and iron oxide nanoparticles used in vivo as imaging agents or adjuvants [8,9,10,11,12,13,14,15,18,19,26,27]. In order to induce magnetic resonance (MR) imaging, NEs should be taken up effectively by cells to different extents and then translated into a different T2-weighted image darkening effect. The Fe concentrations of TC-1 cells treated with NEs were measured by ICP to quantitatively evaluate the cellular uptake (Figure S3). With the increase of NE concentration, Fe concentration in the TC-1 cells gradually increased. After being treated with NEs for 15 min, TC-1 cells can uptake iron-oxide modified NE effectively, which may induce detectable T2 signal changes in vitro.

### 2.2. MR Imaging of Cancer Cells In Vitro

**Figure 4:**
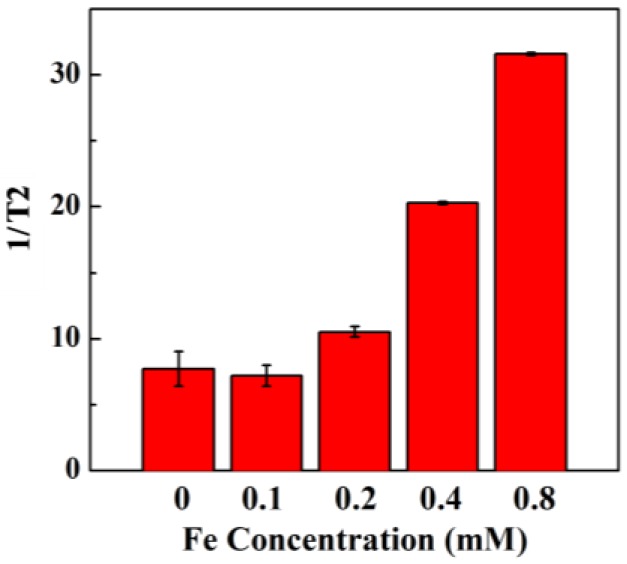
The 1/T2 value of TC-1 cells after treated with PBS, Fe_3_O_4_ modified NEs at different Fe concentrations for 15 min.

In order to demonstrate the MR imaging performance of iron oxide-modified NEs in vitro, TC-1 cells were incubated with different concentrations of iron oxide-modified NEs for 15 min, and were then collected for MR imaging by using a 7T, small animal MR system (Figure 4). It can be clearly seen that the MR image of TC-1 cells becomes darker with the increase of Fe concentration (Figure S4). Subsequently, the 1/T2 rate of iron oxide-modified NE as a function of Fe concentration is quantitatively analyzed. The increase of 1/T2 rate with the increase of Fe concentration in NEs can be clearly seen in Figure 4.

Iron oxide nanoparticles can reduce the intensity of MR images by dephasing the magnetic orientation of the surrounding water protons. The dephasing effect decreases in the observed T2 and T2* times of the infiltrated medium. When higher concentrations of particles are added to the medium, a larger volume of water is subject to this dephasing effect, which then leads to additional reductions in the observed T2 and T2* times.

The results in Figure 4 and Figure S4 were estimated using image slices of the cell pellet, not the supernatant. Thus, the correlation between NE concentration and the estimated 1/T2 rate of the cell pellets suggests that the NE’s did infiltrate the cells and could be applied as a kind of T2 contrast agents for cell MRI imaging.

### 2.3. In Vivo MR Imaging

**Figure 5:**
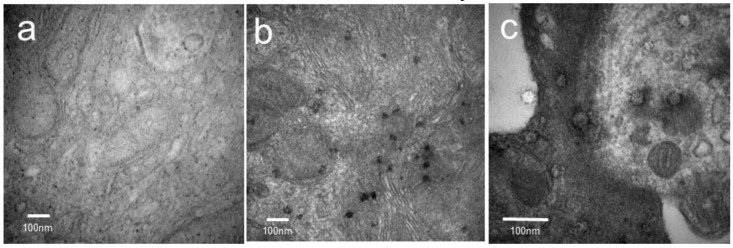
Nasal epithelia structure following the administration of NE and iron oxide-modified NE by TEM. (**a**) Nasal septa from mice treated with NE alone; (**b**) nasal septa from mice treated with iron oxide modified NE; (**c**) nasal septa from mice treated with iron oxide modified NE at the higher resolution.

The ability of iron oxide-modified NE to be visualized after nasal administration was also tested. In these experiments 10 µL (5 µL/nare) of iron oxide-modified NE was administered slowly to the nares, with control mice receiving NE alone. Four hours after administration, TEM studies were done. A signal was detected in the septum of mice administrated with iron oxide-modified NE as darker shadows in TEM imaging (Figure 5b). However, the iron signal could not be observed in the septum of mice treated with NE alone (Figure 5a). At higher resolutions, multiple larger aggregates of iron oxide cores in the mouse septum were recorded (Figure 5c). These findings demonstrate that iron oxide-modified NE is detectable in the mouse nasal septum at least 4 h after administration, and that iron oxide can be used to track NE at least as far as the nasal septum.

One major advantage of the incorporation of iron oxide nanoparticles into NE is that the incorporated iron can be used as a negative contrast agent for MR imaging (MRI) [26,27]. MRI is a very powerful non-invasive method to probe target tissues without risk of radio-damage or other toxic side-effects, and also has advantages of high temporal and spatial resolution that permits the measurement of multiple physiologic parameters with various pulse sequences. MRI can detect the presence of ferromagnetic materials such as iron by using an imaging sequence that is sensitive to the T2* signal decay constant of a tissue of interest. Higher concentrations of iron within the tissue decrease this constant, resulting in reduced signal availability for capture and therefore negative image contrast.

**Figure 6:**
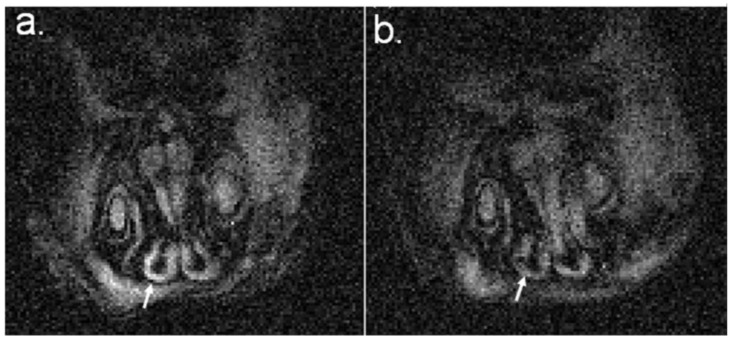
MRI tracking of iron oxide-modified NE in mice. MR images of a mouse sinus both before the administration of iron oxide-modified NE (**a**) and 15–30 min after administration (**b**). The relative loss in image intensity in the sinuses (arrows) from (**a**) to (**b**) is due to an increase in the T2* signal decay rate constant of the sinus. This is thought to be facilitated by the increased concentration of iron in the sinus. The T2* constant for the sinuses is measured to be 8.5 ms before administration 4.4 ms after administration (*p* < 0.05).

In Figure 6, we show two MR images taken using identical MRI sequences during the nasal administration of iron oxide-modified NE to mice. The relative signal loss in the post-treatment image (Figure 6b) compared to the pre-treatment image (Figure 6a) indicates an increased concentration of iron within the sinuses. Further, the T2* constant of the mouse sinus can be estimated by acquiring multiple MR images of the sinus, each with increasing sensitivity, and performing a linear least squares fit [28]. These estimates yield a T2* of 8.5 ± 0.1 ms for the pre-administration case and a T2* of 4.4 ± 0.1 ms for the post-administration case. The significant decrease in T2* after administration is likely due to the increased concentration of iron as the NE is taken up into the sinuses.

## 3. Materials and Methods

### 3.1. Synthesis of Iron Oxide-Modified NE

Iron oxide-modified nanoparticles were prepared using an organic phase synthesis procedure described by Sun et al. [20]. Two mmol of Fe(acac)_3_ was first mixed in 20 mL of phenyl ether with 1,2-hexadecanediol (10 mmol), oleic acid (6 mmol), and oleylamine (6 mmol) under nitrogen flow and vigorous stirring. The mixture was heated to 200 °C for 30 min, and then, under a blanket of nitrogen, heated to reflux (280 °C) for an additional 30 min. After cooling to room temperature, the dark-brown mixture was treated with ethanol under air. The dark-brown material was precipitated from the solution and separated by centrifugation. The precipitated product was dissolved in hexane in the presence of oleic acid and oleylamine and then precipitated again with ethanol. After lyophilisation, iron oxide-modified nanoparticles were dispersed into soybean oil and shaken vigorously. Iron oxide-modified NE was made by mixing the refined soybean oil containing iron oxide nanoparticles, CPC, Tween 80, ethanol and deionized water by a reciprocating syringe pump. The ratio of CPC to Tween 80 was set at 1:6 (one part CPC to six parts Tween 80 by weight) as described previously [14,15].

### 3.2. Characterizations of Iron Oxide-Modified NE

To characterize the morphology and size of Fe_3_O_4_ NPs, TEM was carried out with a JEOL 2010 analytical electron microscope (Tokyo, Japan) operating at 200 kV. Before performing the measurement, the sample was prepared by putting a drop of diluted NP suspension (6 µL) onto a carbon-coated copper grid and dried in air. At least 200 particles in different TEM images were randomly selected and measured by using image software to calculate the size distribution of Fe_3_O_4_ NPs. Hydrodynamic size and zeta potential were measured by a zetasizer. 10% iron oxide-modified NE was diluted to 0.1% NE (*w/v*) with 1 mM HEPES (2-[4-(2-hydroxyethyl)piperazin-1-yl]ethanesulfonic acid) pH 7.4, at room temperature for 2 min before measurement. The structure of iron oxide-modified NE was also illustrated by TEM. Iron oxide-modified NE was mixed with Histo-gel in a ratio of 1:2. Then, a 5 µL aliquot of the mixture was placed on 200 mesh carbon coated copper grids for 10 min, air-dried, rinsed with deionized H_2_O, and stained with 1.0% uranyl acetate for 10 min. Excess stain was removed, and the grids were air-dried. TEM images of iron oxide-modified NE were collected by a Philips CM-100 transmission electron microscope (Andover, MA, USA). Inductively-coupled plasma-high resolution mass spectrometry (ICP-MS, ThemoFisher Scientific, Grand Island, NY, USA) was used to measure the Fe concentration in iron oxide-modified NE. To analysis the distribution of iron oxide in oil phase and water phase, 8.3 µL of 60% iron oxide-modified NE was added to a 2 mL glass tube with 1 mL of H_2_O and mixed thoroughly. After 4 h, the mixture was separated into three phases, with an oil phase on top, followed by an interphase, and then an aqueous phase on the bottom. Iron concentration in the oil, interphase and liquid was measured by ICP-MS.

### 3.3. Cell Culture

TC-1 cell (ATCC, CRL-2785) is a murine epithelial cell line derived from lung epithelial cells from a C57BL/6 mouse. TC-1 cells were continuously cultured in 75 cm^2^ plates with 15 mL of RPMI 1640 media with L-glutamine, containing 10% heat-inactivated FBS (HI-FBS), 1 × nonessential amino acids, 10 mM HEPES, 100 IU penicillin, and 100 µg/mL streptomycin, in an incubator with 5% CO_2_ at 37 °C. The cells were passaged every 3 days when the cells were approaching a density of 80%–90%.

### 3.4. Cytotoxicity Assay and Cell Morphology Observation

The viability of TC-1 cells treated with iron oxide modified NEs in vitro was assessed by XTT assay. Briefly, 1.5 × 10^4^ TC-1 cells were seeded into a 96-well plate per well and incubated 48 h at 37 °C and 5% CO_2_ to bring the cells to confluence. Then the cells were rinsed with 150 µL of 1% BSA/PBS in each well, and the medium was replaced with 100 µL 1% BSA/PBS (control), 1% BSA/PBS containing iron oxide-modified NEs with Fe concentrations at 0.1, 0.2, 0.4, 0.8 mM. After incubated for 15 min, the cells are rinsed with PBS twice, and then 150 µL of fresh medium were added for additional 24 h of incubation. For XTT assay, the medium was aspirated off, and then 100 µL of PBS and 50 µL of XTT-reagent (50 part of XTT labelling reagent and 1 part of electron coupling reagent) were added for 2 h incubation. Read OD as the difference between 492 nm and 690 nm.

Similar to the protocol described above, TC-1 cells were treated with NEs with Fe concentrations ranging from 0 to 0.8 mM for 15 min. Then the cells were washed with PBS twice, trypsinized, resuspended, counted and lysed using an aqua regia solution (nitric acid/hydrochloric acid, *v*/*v* = 3:1). The cellular uptake of the Fe element was measured by ICP-MS.

### 3.5. In Vitro Cell MR Imaging

TC-1 cells were trypsinized and suspended in 1% BSA/PBS in Eppendorf tubes (3 × 10^6^ cells per tube), and incubated with iron oxide loaded NE at Fe concentrations of 0.1, 0.2, 0.4, and 0.8 mM for 15 min. The cells were centrifuged, washed by PBS 2 times, and resuspended in 100 µL PBS in 0.7 mL Eppendorf tubes before MR imaging. The T2-weighted MR imaging of the cell was carried out by using a 7T Agilent MR Scanner (Agilent, Santa Clara, CA, USA). Scans were conducted using a multi-echo-multi slice sequence. The imaging parameters were: TR: 7 s, echo spacing: 10 ms, number of echoes: 12, FOV: 12 *×* 8 *×* 0.2 cm, Matrix: 120 *×* 80 *×* 14, Spectral Width: 100 kHz, Averages: 1, Imaging Time: 8.33 min.

After imaging, the 1/T2 relaxation rate was estimated by performing a non-linear, least squares fit to the equation:

(1) $$y_{i}=S_{o}+S_{x}e^{-TE_{i}/T2}$$

where *y_i_* is the signal of the NE tubes acquired at the echo time *TE_i_* , and *S*_0_ and *S_x_* are terms that account for non-idealities in the experiment and are computed along with 1/T2 during the fitting operation.

### 3.6. In Vivo TEM

Eight-week-old, CD-1 female mice were purchased from Charles River Labs and used for an in vivo TEM study. 5 µL of 20% of iron oxide modified NE was administered slowly to each nare, with control mouse receiving unmodified NE. Four hours after administration, mouse nasal septa were harvested and fixed in 3% paraformaldehyde and 2.5% glutaraldehyde in 0.1 M Soensen*’*s buffer, pH 7.4, followed by post fixation in 1% osmium tetroxide in 0.1 M Sorensen*’*s buffer. After dehydration, the septa were infiltrated in Epon 812. The embedded tissue blocks were sectioned. Images were collected with a Philips CM-100 transmission electron microscope at 100 kV.

### 3.7. Magnetic Resonance Imaging (MRI)

MR imaging of the in vivo uptake of the iron oxide modified NE was performed in eight-week-old female outbred CD-1 mice. All procedures were approved by the University Committee on the Use and Care of Animals at the University of Michigan. Mice were anesthetized under inhalation of isoflurane through the course of the experiment. For pre-administration images, mouse was placed in a 7-Tesla small animal MRI scanner (Agilent, Walnut Creek, CA, USA) with a 2 cm diameter surface coil centered on the sinus. The sinuses were then imaged using a gradient-recalled-echo pulse sequence. The imaging parameters were: TR: 500 ms, FOV: 1 *×* 1 *×* 0.1 cm, Matrix: 128 *×* 128 *×* 5, Spectral Width: 50 kHz, Averages: 8, Imaging Time: 8.5 min. This sequence was repeated three times with echo times of 4.3 ms, 6.3 ms and 8.3 ms.

After pre-administration imaging the mouse was removed from the MRI scanner. 10 µL (5 µL per nare) of iron oxide-modified NE was delivered nasally and the mouse was placed back in the scanner. Images of the mouse sinus were then acquired with an identical imaging sequence except that the echo times were shortened to 4.3, 5.3 and 6.3 ms in order to compensate for the increased signal loss caused by the introduction of iron to the nasal passages.

After imaging, the T2* constants of the pre- and post-administration images were estimated by selecting signal from the nasal passages and performing a linear least squares fit to the equation:

(2) $$\ln(y_{i})=-TE_{i}/T2^{*}+\ln(S_{o})$$

where *y_i_* is the signal of the sinus in the image taken at echo time *TE_i_*, and *S*_0_ is the theoretical signal level that would be observed if the MRI signal decay processes were not present.

### 3.8. Statistical Analysis

The values given are presented as mean ± SD. Statistical analysis was performed using one-way analysis of variance (ANOVA) followed by Student’s *t-*test. *p* < 0.05 was considered significant.

## 4. Conclusions

Altogether, both TEM and MRI demonstrate that NEs modified by Fe_3_O_4_ nanoparticles can be used to study NE administration and uptake in vivo, and confirm that NEs are taken up into the nasal tissue. Future work can potentially track the migration of the iron oxide-modified NE into the regional lymph nodes and thymus using a similar technique.

## Supplementary Materials

The following are available online at http://www.mdpi.com/2079-4991/6/12/223/s1.
