# Supplementary material for: Modified Nanoemulsions with Iron Oxide for Magnetic Resonance Imaging
Source: Nanomaterials (Basel). 2016 Nov 25;6(12):223. doi: 10.3390/nano6120223 (PMC5302717; doi:10.3390/nano6120223)
Supplement: Supplementary file 1 [file nanomaterials-06-00223-s001.pdf]

Supplementary Materials: Modified Nanoemulsion with Iron Oxide for Magnetic Resonance Imaging

Yongyi Fan, Rui Guo, Xiangyang Shi, Steven Allen, Zhengyi Cao, James R. Baker and Su He Wang

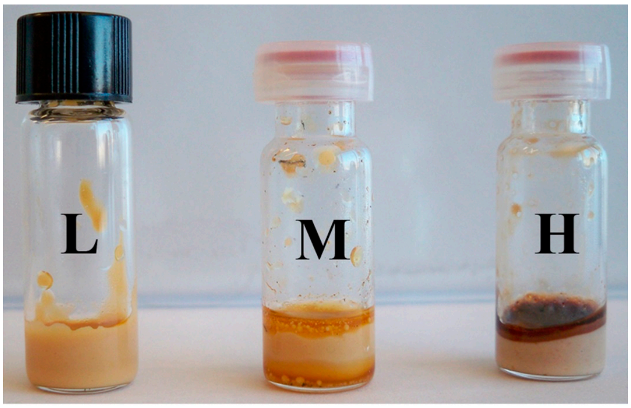

	Size (nm)	ZP (mv)
Modified NE-L	521.1	9.64
Modified NE-M	704.8	9.66
Modified NE-H	1469.0	23.7

Figure S1. Stability of iron oxide-modified NE with different concentrations of iron oxide nanoparticles. L: low concentration (30 µg/µL); M: moderate concentration (35 µg/µL); H: high concentration (40 µg/µL). For each formulation the size of the droplets and the zeta potential are shown.

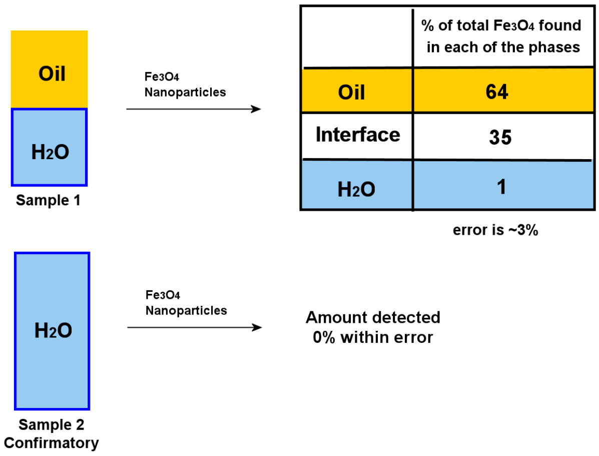

Figure S2. The distribution of Fe_3O_4 in oil phase and water phase of iron oxide-modified nanoemulsion.

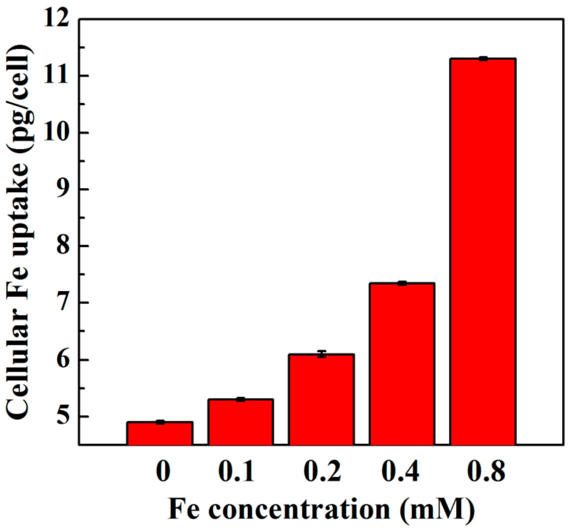

Figure S3. The Fe uptake by TC-1 cells treated with Fe_3O_4 modified NEs at the Fe concentration of 0–0.8 mM for 15 min.

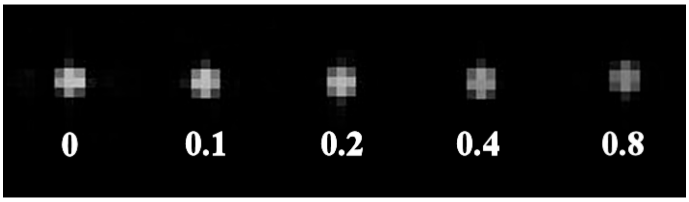

Figure S4. The MR image of TC-1 cells with 0–0.8 mM of iron oxide-modified NEs.

© 2016 by the authors; licensee MDPI, Basel, Switzerland. This article is an open access article distributed under the terms and conditions of the Creative Commons by Attribution (CC-BY) license (http://creativecommons.org/licenses/by/4.0/).
